# The Effects of Specific Gut Microbiota and Metabolites on IgA Nephropathy—Based on Mendelian Randomization and Clinical Validation

**DOI:** 10.3390/nu15102407

**Published:** 2023-05-22

**Authors:** Fang Wang, Ning Li, Siming Ni, Yu Min, Kang Wei, Hongbin Sun, Yuqi Fu, Yalan Liu, Dan Lv

**Affiliations:** 1 Institute of Nephrology, Liyang Hospital of Chinese Medicine, Liyang 213300, China; 2 Jiangsu Province Hospital of Traditional Chinese Medicine, Nanjing University of Chinese Medicine, Nanjing 210000, China; 3 Institute of Nephrology, Southeast University School of Medicine, Nanjing 210009, China; 4 Department of Biotherapy and National Clinical Research Center, West China Hospital, Sichuan University, Chengdu 610041, China; 5 Yangzhou People’s Hospital, Yangzhou University, Yangzhou 225000, China

**Keywords:** Mendelian randomization, intestinal flora, gut metabolites, genetic, causal effect

## Abstract

Background: Although recent research suggests that alterations in gut microbiota and metabolites play a critical role in the pathophysiology of immunoglobulin A nephropathy (IgAN), the causal relationship between specific intestinal flora and metabolites and the risk of IgAN remains unclear. Method: This study employed Mendelian randomization (MR) to investigate the causal association between gut microbiota and IgAN. To explore potential associations between gut microbiota and various outcomes, four MR methods were applied: inverse variance weighted (IVW), MR-Egger, weighted median, and weighted mode. If the results of the four methods are inconclusive, we prefer the IVW as the primary outcome. Additionally, MR-Egger, MR-PRESSO-Global, and Cochrane’s Q tests were used to detect heterogeneity and pleiotropy. The stability of MR findings was assessed using the leave-one-out approach, and the strength of the causal relationship between exposure and outcome was tested using Bonferroni correction. Additional clinical samples were utilized to validate the results of Mendelian randomization, and the outcomes were visualized through an ROC curve, confusion matrix, and correlation analysis. Result: This study examined a total of 15 metabolites and 211 microorganisms. Among them, eight bacteria and one metabolite were found to be associated with the risk of IgAN (*p* < 0.05). The Bonferroni-corrected test reveals that only Class. Actinobacteria (OR: 1.20, 95% CI: 1.07–1.36, *p* = 0.0029) have a significant causal relationship with IgAN. According to Cochrane’s Q test, there is no substantial heterogeneity across different single-nucleotide polymorphisms (*p* > 0.05). Furthermore, MR-Egger and MR-PRESSO-Global tests (*p* > 0.05) showed no evidence of pleiotropy. No reverse causal association was found between the risk of IgAN and microbiota or metabolites (*p* > 0.05). Clinical specimens demonstrated the effectiveness and accuracy of Actinobacteria in distinguishing IgAN patients from those with other glomerular diseases (AUC = 0.9, 95% CI: 0.78–1.00). Additionally, our correlation analysis revealed a potential association between Actinobacteria abundance and increased albuminuria (r = 0.85) and poorer prognosis in IgAN patients (*p* = 0.01). Conclusion: Through MR analysis, we established a causal link between Actinobacteria and the incidence of IgAN. Moreover, clinical validation using fecal samples indicated that Actinobacteria might be associated with the onset and poorer prognosis of IgAN. This finding could provide valuable biomarkers for early, noninvasive detection of the disease and potential therapeutic targets in IgAN.

## 1. Background

Immunoglobulin A nephropathy (IgAN), the most prevalent primary glomerular disease worldwide [1], is also the leading cause of end-stage renal disease (ESRD) [2]. Presently, the treatment of IgAN relies primarily on renin-angiotensin-aldosterone system (RAAS) inhibitors and glucocorticoids [3]. However, the increased side effects and poor sensitivity experienced by some patients make IgAN treatment a challenging issue. Consequently, finding a better approach to prevent and intervene in IgAN has become a global focus for nephrologists.

In recent years, modulating the crosstalk between the gut and kidney axis has emerged as a promising strategy for treating IgAN [4,5,6]. It is believed that the pathogenesis of IgAN may be triggered by intestinal infections that activate the intestinal mucosal immune system [7]. The overproduction of abnormally glycosylated IgA antibodies by intestinal mucosal immune cells leads to a cascade of subsequent multiple immune attacks [8,9,10]. It has been demonstrated that gut microbiota and metabolites play a crucial role in inducing IgAN mucosal immunity [11,12,13]. Metabolites released by dysregulated flora, such as indoxyl sulfate, *p*-cresyl sulfate, indole-3-acetic acid, trimethylamine N-oxide (TMAO), and phenylacetylglutamine, may compromise the integrity of the intestinal barrier [11,14]. This makes the intestinal tract more susceptible to bacterial invasion, which in turn triggers the immune system in the intestinal mucosa. Some studies focusing on modulating microbiota and metabolites have reported reduced clinical phenotypes in IgAN mice [15,16]. Therefore, altering gut microbiota or metabolites may become a novel therapeutic target in IgAN.

While targeting bacteria and metabolites to treat IgAN may become an emerging therapeutic modality, identifying the specific gut microbiota and metabolites that have a causal relationship with IgAN remains challenging due to the complexity of the human gut microbiota. Previous studies primarily relied on animal models or observational cross-sectional data, and although some associations were suggested, none could elucidate the causal relationship. This makes it difficult to precisely target the flora for IgAN treatment. Notably, gene prediction based on GWAS data appears to hold potential in helping us solve this puzzle. Mendelian randomization (MR) [17] is based on Mendel’s second law (the law of independent assortment of genes). The causal relationship between risk factors and outcomes can be demonstrated at the gene level without the interference of confounding variables by leveraging single-nucleotide polymorphisms (SNPs) associated with the clinical phenotype. The causal relationship determined by the MR method is more accurate compared to traditional observational research. This can help us better identify the causal relationship between specific human intestinal flora and IgAN, which holds significant implications for more precise targeted regulation of bacteria to prevent IgAN. Therefore, our study aims to investigate the causal effect of specified gut microbiota and metabolites on IgAN, which may provide valuable biomarkers for early, noninvasive disease diagnosis and potential therapeutic targets for IgAN.

## 2. Method

### 2.1. Data Source of Exposure and Outcome

We selected 211 intestinal microflora (131 genera, 35 families, 20 orders, 16 classes, and 9 phyla) and 12 metabolites (i.e., beta-hydroxybutyric acid, betaine, TMAO, carnitine, choline, glutamate, kynurenine, phenylalanine, propionic acid, serotonin, tryptophan, tyrosine) as exposure factors. The GWAS statistics data of microbiota primarily came from a large-scale multi-ethnic GWAS meta-analysis of 18,340 individuals from 24 cohorts, which recorded 211 gut microbiota and 122,110 related SNPs [18], while the data of metabolites were derived from a summary data of the human metabolome in 2076 participants from the Framingham Heart Study [19]. IgAN was considered the primary outcome in this study. The IgAN statistics data (https://www.ebi.ac.uk/gwas/studies/GCST90018866, accessed on 15 August 2022) came from a meta-analysis of 628,000 cross-population samples from the UK Biobank and FinnGen [20].

### 2.2. The Selection of Instrumental Variables

Our study satisfied three assumptions of MR analysis. First, the single-nucleotide polymorphisms (SNPs) selected for MR analysis need to be strongly associated with the selected microbiota or metabolites. In our research, to ensure enough instrumental variables were included, we chose SNPs with *p*-values below the locus-wide significance level (1 × 10^−5^) for analysis. To ensure the robust association between instrumental variables and exposure factors, we excluded instrumental variables with F values (formula: (R2/(R2 − 1))×((N − K − 1)/K)) < 10. Second, the chosen instrumental variables must meet an independence test. The SNP linkage disequilibrium value (r2) was set to 0.001 and the genetic distance to 10,000 kb to eliminate linkage disequilibrium impact and maintain the independence of selected instrumental variables. Those with an EAF value of less than 0.01 were also excluded. Third, instrumental variables should not be associated with outcomes. We excluded the instrumental variables associated with IgAN (*p* < 0.05). The Phenoscanner [21] was also used to exclude instrumental variables associated with various confounding factors (e.g., tonsil infection, hypertension, and some autoimmune diseases). The aforementioned list of instrumental variables screening ensures the validity of our study’s findings.

### 2.3. Mendelian Randomization Analysis

Inverse variance weighting (IVW), MR-Egger, weighted median, and weighted mode were used to examine the causal effects. IVW is a time-honored method for combining the Wald ratio estimates of all relevant instrumental variables. This strategy is analogous to using weighted linear regression to probe the relationships between the instrumental factors and the outcome. The intercept of the instrumental variables is constrained to zero. IVW can obtain unbiased estimates of the status without horizontal pleiotropy. Under the premise of InSIDE, the MR-Egger method primarily demonstrates the dosage relationship between instrumental variables and outcomes while accounting for some pleiotropy. The type I error rate can be lowered using the weighted median method, which also allows for the possibility of invalidity for some specific genetic variants. The weighted mode approach remains valid when the vast majority of instrumental variables with identical causal estimates are valid, even if certain instrumental variables do not satisfy the requirements of the MR technique for causal inference. If the results of these methods are inconsistent, we give priority to IVW as the main result.

The MR-Egger and MR Pleiotropy RESidual Sum and Outlier (MR-PRESSO) tests were employed to check for horizontal pleiotropy and outliers, respectively. Using MR-Egger [22], we may make a first judgment as to whether or not there is horizontal pleiotropy. If the *p*-value was greater than 0.05, then it was deemed that horizontal pleiotropy was not present in a statistically significant way. When comparing MR-Egger to MR-PRESSO [23], the latter has improved precision in detecting horizontal pleiotropy and outliers. Instrument variable heterogeneity was examined using Conchrane’s Q test. The results of the outliers and the consistency of the study were analyzed using a leave-one-out sensitivity analysis. We also utilized the Bonferroni-corrected test, which takes into account the number of bacteria in each attribute group, to establish a more robust causal association (genera: 0.05/131 (3.81 × 10^−4^), families: 0.05/35 (1.4 × 10^−3^), orders: 0.05/20 (2.5 × 10^−3^), classes: 0.05/16 (3.1 × 10^−3^) and phyla: 0.05/9 (5.5 × 10^−3^)), and metabolites (0.05/15 (3.3 × 10^−3^)). We performed additional reverse causal analysis to investigate whether IgAN would have an effect on the abundance of the identified microorganisms. It was determined that a causal effect was nominal when the *p*-value was between 0.05 and the corrected value. The statistical analyses were performed using R software version 4.1.2 (https://www.rproject.org/ accessed on 15 August 2022).

### 2.4. Clinical Validation

#### 2.4.1. Study Population

We recruited a total of 10 patients with renal biopsy-confirmed IgAN, 5 patients with other types of glomerular disease, and 10 matched healthy controls who had not received corticosteroid, immunosuppressive, or antibiotic therapy prior to sampling. All participants were recruited from the Jiangsu Province Hospital of Chinese Medicine between May 2021 and August 2022. To ensure comparability between groups, we maintained consistency across all factors except kidney disease, including sex, age, medical history, medication use, lifestyle, and dietary habits. We excluded patients with intestinal disorders and autoimmune diseases from the study. Healthy controls were recruited from the Physical Examination Center of Jiangsu Hospital of Traditional Chinese Medicine. The study was reviewed and approved by the Ethics Committee of Jiangsu Provincial Hospital of Traditional Chinese Medicine and complied with the Declaration of Helsinki.

#### 2.4.2. Sample Collection

The participants were provided with a special stool collection container and instructed to collect 5 g of feces immediately after defecation, which was then placed in a professional stool DNA preservation solution. The samples were stored at a temperature of −80 °C. Once a sufficient number of samples were collected, they were sent to a professional testing institution (Kechuang Biotechnology Co., Ltd., Shenzhen, China) for analysis.

#### 2.4.3. DNA Extraction and Sequencing of Samples

In this study, we employed the QIAamp DNA Stool Mini Kit method, utilizing silicone membrane technology to extract DNA from 25 frozen stool samples. Subsequently, all samples were assessed for quality and quantity via 3 µL test 1.2% agarose gel electrophoresis and qualitative evaluation of the extracted DNA. The target region and fusion primers were designed to meet the sequencing platform’s requirements, and PCR amplification was carried out using a two-step method. The PCR products were then recovered through 2% agarose gel and purified using the AxyPrep DNA Gel Extraction Kit. The resulting purified products were further eluted and analyzed using 2% agarose gel electrophoresis detection, and FTC-3000TM real-time PCR Quantification was employed for detection. All samples were high-throughput sequenced using the Illumina sequencing platform, followed by microbiome analysis.

#### 2.4.4. Statistical Analysis

Initially, we assessed whether the necessary variables adhered to a normal distribution and exhibited homogeneity of variance. If these criteria were met, we proceeded with a paired t-test to evaluate differences between groups. For comparisons involving multiple groups, we employed a one-way ANOVA test. In cases where these assumptions were not satisfied, we resorted to a Wilcoxon rank sum test for analysis or utilized the Kruskal-Wallis test to examine variables across various groups. This approach ensures a comprehensive, accurate, and elegant assessment of the data. For counting data, we used a chi-square test for comparison. The area under the receiver operating characteristic curve (AUC-ROC) was used to detect the distinguishing ability of biomarkers. The Pearson correlation coefficient was used to describe the correlation between measurement data. All statistical analyses were performed using R4.2.0, with *p*-values less than 0.05 indicating statistical significance.

## 3. Results

### 3.1. The Selection of Instrumental Variables

The instrumental variables for 211 distinct microorganisms were individually screened. A total of 14,587 instrumental variables (Supplementary Table S1) reached the locus-wide significance level (*p* < 10^−5^). 3678 instrumental variables were excluded due to linkage disequilibrium. The final dataset included 3631 instrumental variables from 211 different microorganisms after removing variables with weak connections to the exposure factors and variables that might be associated with confounding factors of outcomes. Since there were not enough instrumental variables for screening metabolites that met the locus-wide significance level, we increased the *p*-value threshold to 5 × 10^−5^ (Supplementary Table S2), ultimately retaining a total of 514 instrumental variables.

### 3.2. Two Samples MR Analysis

#### 3.2.1. Gut Microbiota

**Figure 1:**
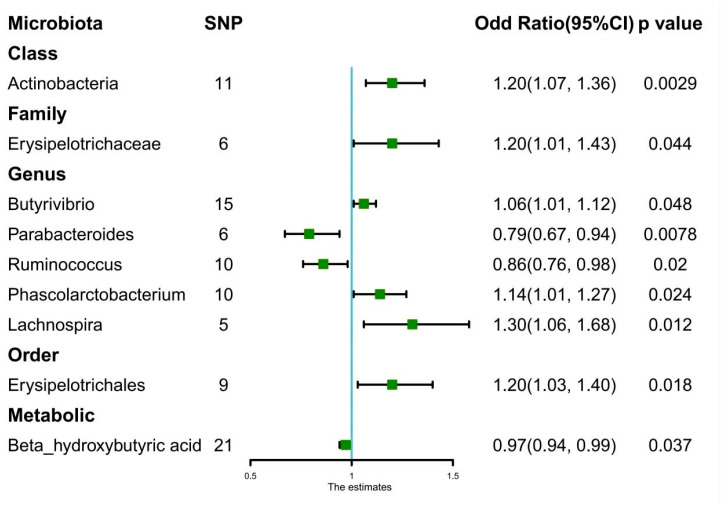
Forest plots of estimates (Odds Ratio) identified with inverse variance weighted. Abbreviation: SNP: single-nucleotide polymorphisms.

**Figure 2:**
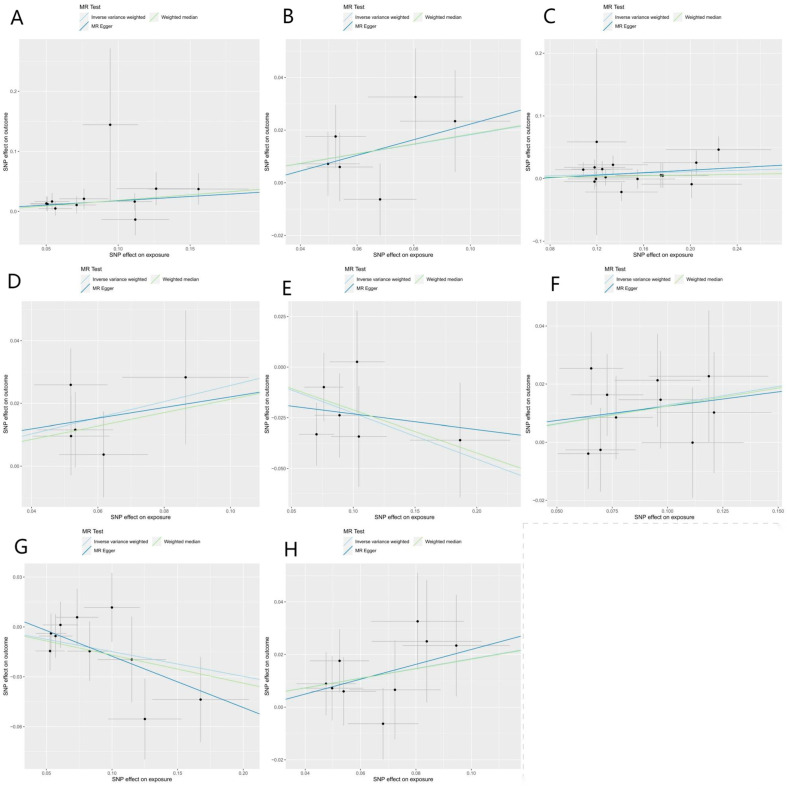
Scatter plots of causal estimates from genetically predicted microbiota on immunoglobulin A nephropathy. (**A**) Class Actinobacteria; (**B**) Family Erysipelotrichaceae; (**C**) Genus Butyrivibrio; (**D**) Genus Lachnospira; (**E**) Genus Parabacteroides; (**F**) Genus Phascolarctobacterium; (**G**) Genus Ruminococcus; (**H**) Order Erysipelotrichales.

**Figure 3:**
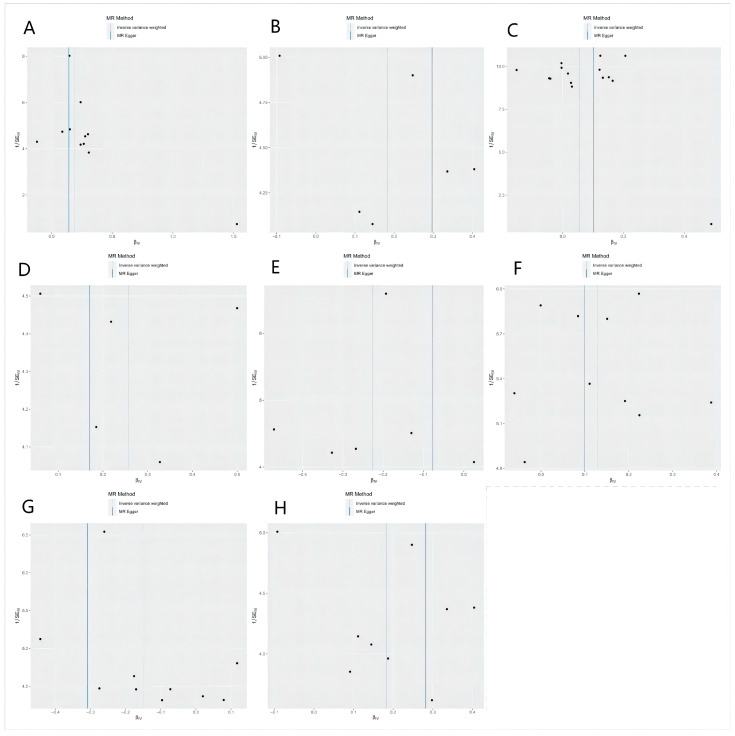
Funnel plots of causal estimates from genetically predicted microbiota on immunoglobulin A nephropathy. (**A**) Class Actinobacteria; (**B**) Family Erysipelotrichaceae; (**C**) Genus Butyrivibrio; (**D**) Genus Lachnospira; (**E**) Genus Parabacteroides; (**F**) Genus Phascolarctobacterium; (**G**) Genus Ruminococcus; (**H**) Order Erysipelotrichales.

**Figure 4:**
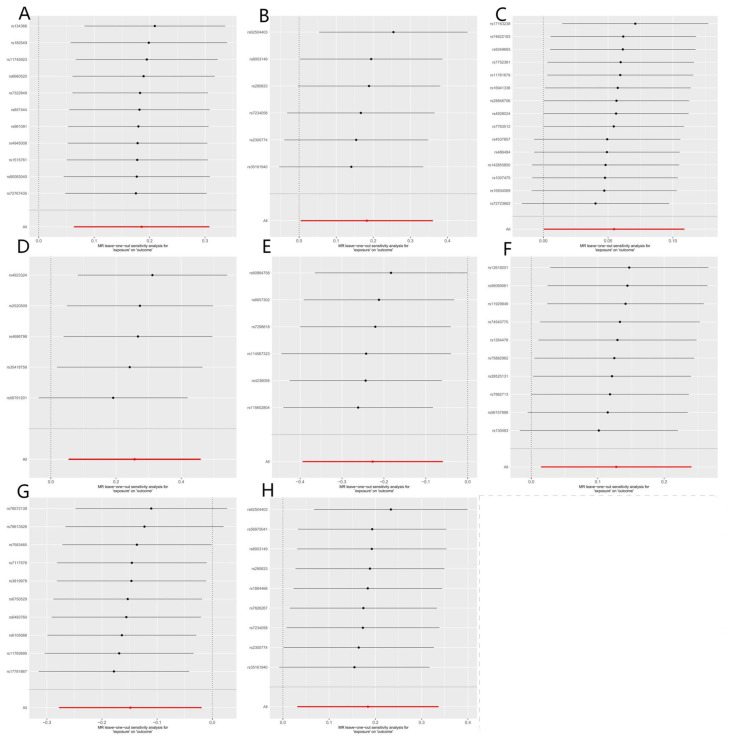
Leave-one-out plots of causal estimates from genetically predicted microbiota on immunoglobulin A nephropathy. (**A**) Class Actinobacteria; (**B**) Family Erysipelotrichaceae; (**C**) Genus Butyrivibrio; (**D**) Genus Lachnospira; (**E**) Genus Parabacteroides; (**F**) Genus Phascolarctobacterium; (**G**) Genus Ruminococcus; (**H**) Order Erysipelotrichales.

A total of eight bacteria (Figure 1) were identified as being associated with IgAN. Among them, Class Actinobacteria (OR: 1.20, 95% CI: 1.07–1.36, *p* = 0.0029), Family Erysipelotrichaceae (OR: 1.20, 95% CI: 1.01–1.43, *p* = 0.044), Genus Butyrivibrio (OR: 1.06, 95% CI: 1.01–1.12, *p* = 0.048), Genus Phascolarctobacterium (OR: 1.14, 95% CI: 1.01–1.27, *p* = 0.024), Genus Lachnospira (OR: 1.30, 95% CI: 1.06–1.68, *p* = 0.012), and Order Erysipelotrichales (OR: 1.20, 95% CI: 1.03–1.40, *p* = 0.018) were associated with a higher risk of IgAN, according to IVW analysis. In contrast, Genus Parabacteroides (OR: 0.79, 95% CI: 0.67–0.94, *p* = 0.0078) and Genus Ruminococcus (OR: 0.86, 95% CI: 0.76–0.98, *p* = 0.02) were linked to a lower risk of IgAN. The weighted median method (Table 1) revealed that Class Actinobacteria (OR: 1.20, 95% CI: 1.03–1.41, *p* = 0.024) was associated with a higher risk of IgAN. The weight mode method produced a similar result (Class Actinobacteria: OR: 1.27, 95% CI: 1.05–1.53, *p* = 0.036). The MR-Egger, MR-PRESSO test (Supplementary Table S3), and scatter plot (Figure 2) demonstrated that there was no horizontal pleiotropy or outliers (*p* > 0.05). Cochrane’s Q test (Supplementary Table S3) and funnel plot (Figure 3) indicated that no significant heterogeneity was found among the selected SNPs (*p* > 0.05). Nonetheless, the leave-one-out method (Figure 4) showed that certain individual SNPs might account for the majority of the favorable findings.

#### 3.2.2. Gut Metabolites

Only Beta-hydroxybutyric acid (OR: 0.97, 95% CI: 0.94–0.99, *p* = 0.037) was found to be associated with a lower risk of IgAN (Figure 1), according to IVW analysis. However, weight mean (OR: 0.97, 95% CI: 0.93–1.01, *p* = 0.17), weight mode (OR: 0.98, 95% CI: 0.93–1.02, *p* = 0.30), and MR-Egger analysis (OR: 0.98, 95% CI: 0.92–1.05, *p* = 0.57) did not show a significant effect (Table 1). MR-Egger, MR-PRESSO test (Supplementary Table S3), and scatter plot (Supplementary Figure S1) indicated that there was no horizontal pleiotropy (*p* > 0.05). Cochrane’s Q test (Supplementary Table S3) and funnel plot (Supplementary Figure S2) demonstrated that no significant heterogeneity was found in the selected SNPs (*p* > 0.05). Nonetheless, the leave-one-out method (Supplementary Figure S3) revealed that the majority of the positive results might be attributed to a few distinct SNPs.

#### 3.2.3. Bonferroni-Corrected Test and Reverse Causality

Only Class Actinobacteria (OR: 1.20, 95% CI: 1.07–1.36, *p* = 0.0029) satisfied the Bonferroni-corrected test after analyzing the genetic association between 211 microbiota and IgAN (Figure 1). No metabolites achieved significant causal effects on IgAN (*p* > 3.3 × 10^−3^). Table 1 displays the causal relationship between intestinal flora and various outcomes. The reverse causal analysis (Supplementary Table S4) did not show a significant causal effect of IgAN on microbiota or metabolites (*p* > 0.05).

### 3.3. Clinical Validation

#### 3.3.1. Baseline Characteristic

A total of 25 patients were included in the study (Table 2), consisting of 10 patients diagnosed with IgAN, 10 healthy controls, and 5 patients with other glomerular diseases, including Membranous nephropathy (n = 2), Lupus nephropathy (n = 2), and Focal segmental glomerulosclerosis (n = 1). The three groups did not display significant differences in age, sex, systolic blood pressure, body mass index, and serum albumin level (*p* > 0.05). However, patients with IgAN and other glomerular diseases exhibited lower estimated glomerular filtration rate (eGFR), higher albuminuria levels, lower hemoglobin, and higher incidence of hypertension compared with healthy controls (*p* < 0.05). Based on these results, we conducted pairwise comparisons of *Actinobacteria* abundance between the three groups to investigate the potential association between *Actinobacteria* and IgAN.

#### 3.3.2. Class Actinobacteria Is Associated with IgAN

**Figure 5:**
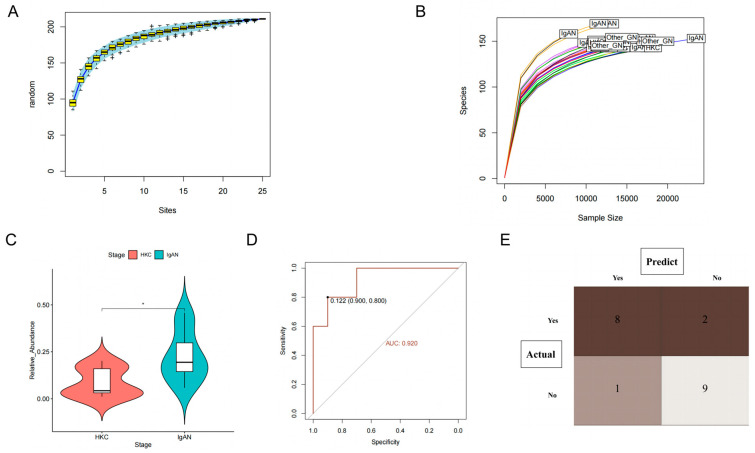
Clinical validation of Actinobacteria in the identification of immunoglobulin A nephropathy. (**A**) Species accumulation curve for clinical samples; (**B**) Dilution curves of sequencing depth and species richness; (**C**) Differences in Actinobacteria abundance between IgAN and healthy controls; (**D**) The receiver operating characteristic (ROC) of Actinobacteria (compare with healthy control); (**E**) The confusion matrix of Actinobacteria discriminating abilities (compare with healthy control).

Based on the species accumulation curves presented in Figure 5A and the dilution curve shown in Figure 5B, it is evident that the study’s sample size and sequencing depth meet the necessary criteria. As a result, we aim to investigate the possible involvement of *Actinobacteria* in the development and advancement of IgAN. Our first objective was to determine whether *Actinobacteria* could be utilized to differentiate between IgAN patients and healthy controls. To accomplish this, we utilized a paired sample t-test and observed that patients diagnosed with IgAN (Figure 5C) had a significantly higher abundance of *Actinobacteria* compared to healthy controls (*p* < 0.05). The AUC was calculated to be 0.92 (95% CI: 0.80–1.00), indicating that this microflora possessed an exceptional ability to distinguish IgAN from healthy controls (Figure 5D). Additionally, we constructed a ROC curve to determine the optimal cutoff value for *Actinobacteria* identification, which was determined to be 0.122. As a result, *Actinobacteria* richness greater than 0.122 was classified as IgAN patients, and the results were visualized through a confusion matrix (Figure 5E). Our findings demonstrated that the sensitivity, specificity, and accuracy of this biomarker were 0.89, 0.82, and 0.85, respectively. These results indicate that this marker can accurately differentiate between the healthy control group and IgAN patients.

#### 3.3.3. Class Actinobacteria Can Be Used to Distinguish IgAN from Other

##### Glomerular Diseases

**Figure 6:**
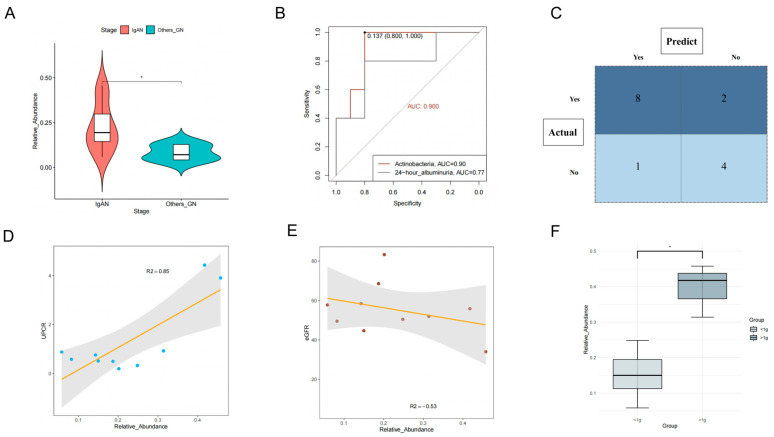
The association of Actinobacteria abundance with the progression of immunoglobulin A nephropathy. (**A**) Differences in Actinobacteria abundance between IgAN and other glomerular diseases; (**B**) The receiver operating characteristic (ROC) of Actinobacteria (compare with other glomerular diseases); (**C**) The confusion matrix of Actinobacteria discriminating abilities (compare with other glomerular diseases); (**D**) Scatter plot of the association between Actinobacteria and 24-h albuminuria; (**E**) Scatter plot of the association between Actinobacteria and estimated glomerular filtration rate (eGFR); (**F**) Differences in Actinobacteria abundance between high-risk (>1 g/24 h albuminuria) and low-risk group (<1 g/24 h albuminuria). *: *p* < 0.05.

Clinicians are actively seeking noninvasive methods to differentiate IgAN from other types of glomerular diseases. To address this issue, we conducted a comparative analysis of the fecal microbiota in IgAN patients and those diagnosed with other glomerular diseases. Our results showed (Figure 6A) that the relative abundance of *Actinobacteria* in the fecal microbiota of IgAN patients was significantly higher than that of patients with other glomerular diseases (*p* < 0.05). The AUC value for distinguishing IgAN from other glomerular diseases was 0.90 (95% CI: 0.78–1.00). Notably, we compared the differential ability of 24-h urinary protein (Figure 6B), which had an AUC of 0.78 (95% CI: 0.49–0.98), indicating that proteinuria alone was insufficient for distinguishing IgAN from other glomerular diseases. Using the cutoff value, we constructed a confusion matrix (Figure 6C), and our research results indicated that this microbiota biomarker displayed an accuracy, sensitivity, and specificity of 0.80, 0.89, and 0.67, respectively. This suggests that this biomarker still exhibits promising performance in distinguishing IgAN from other glomerular diseases. 

#### 3.3.4. Class Actinobacteria Is Associated with the Progression of IgAN

To further explore the potential clinical relevance of Actinobacteria in IgAN patients, we investigated the correlation between Actinobacteria abundance and various indicators, including proteinuria, eGFR, and prognosis. Using the Pearson index to study the correlation among these indicators (Figure 6D), we found that an increased abundance of Actinobacteria was significantly associated with higher albuminuria levels (r = 0.85, *p* = 0.005). Although we observed a trend of increased Actinobacteria richness and decreased eGFR (r = −0.53), the correlation coefficient (Figure 6E) was not significant (*p* = 0.108). Notably, in non-IgAN glomerular diseases, Actinobacteria showed no significant correlation (Supplement Figure S4) with proteinuria and eGFR (*p* > 0.05). This suggests that Actinobacteria may be a unique pathogenic factor specific to IgAN progression.

IgAN patients with 24-h albuminuria levels > 1 g tend to have poorer renal outcomes, and we grouped them accordingly. The results (Figure 6F) demonstrated that the group with > 1 g/24 h albuminuria had a higher percentage of Actinobacteria (*p* = 0.01), suggesting that increased Actinobacteria abundance may be associated with a poorer prognosis in IgAN patients. In addition, we conducted a correlation analysis between the relative abundance of Actinobacteria and the pathological scores (MEST-C) of IgAN patients. Based on the median abundance (>20%), patients were divided into high-abundance and low-abundance groups. Although there were no significant differences (Supplementary Table S5) in the proportions of pathological grades between the groups (*p* > 0.05), a bar chart analysis (Supplement Figure S5) of the proportions revealed that the high-abundance group tended to have more severe pathological grades. This also suggests that Actinobacteria may be associated with more severe kidney injury in IgAN patients.

## 4. Discussion

Emerging evidence demonstrates that the interplay between the gut and kidney axis plays a pivotal role in the pathogenesis of IgA nephropathy (IgAN) [24,25]. Targeting the intestinal flora and metabolites may interfere with the intestinal mucosal immune system, thereby preventing and delaying the progression of IgAN [26,27]. However, the causal effect of specific gut microbiota and metabolites on IgAN remains elusive. To the best of our knowledge, our study is the first large-scale comprehensive Mendelian randomization (MR) investigation that explores the causality between specific gut microbiota and IgAN. Most studies examining the association between intestinal microorganisms and IgAN have relied on animal models or cross-sectional data, which fail to elucidate causality. Employing genetic prediction, our study reveals that several specific bacteria and metabolites play a crucial role in the pathogenesis of IgAN. This finding may provide valuable biomarkers for early, noninvasive diagnosis and potential therapeutic targets for IgAN.

In this study, we genetically predicted the causal relationship between 211 bacteria and IgAN. According to MR analysis, we identified a total of eight associations between gut microbiota and chronic kidney disease (CKD). Among them, Class Actinobacteria exhibited a strong causal relationship with a higher risk of IgAN (OR: 1.20, 95% CI: 1.07–1.36, *p* = 0.0029). Furthermore, our research results show no significant heterogeneity or horizontal pleiotropy, suggesting stability. Notably, clinical sample validation also found that a high abundance of Actinobacteria can distinguish IgAN from other glomerular diseases (*p* < 0.05). As a gram-positive bacterium, Actinobacteria can produce numerous secondary metabolites and have been associated with various immune kidney diseases [28,29]. Nonetheless, the abundance of Actinobacteria in IgAN patients remains a controversy. Most cross-sectional study results lean towards higher levels of Actinobacteria in the feces of IgAN patients, while Zhong et al. [26] reported significantly lower abundance in IgAN patients. A bibliometric study [13] found no significant difference in the richness of feces between IgAN patients and healthy controls. This discrepancy may be due to the considerable heterogeneity of the included literature. Our study provides genetic evidence for the causative relationship between Actinobacteria and IgAN, which has not been demonstrated in previous cross-sectional studies. Interestingly, our clinical sample validation revealed that Actinobacteria was associated with higher albuminuria (r = 0.85) and poor prognosis in IgAN patients. We speculate that the observed phenomenon may result from the accumulation of microbiota levels, leading to increased production of metabolic toxins. The continuous buildup of these toxins exacerbates inflammation and results in elevated levels of albuminuria. These findings suggest that a high abundance of Actinobacteria could serve as a non-invasive diagnostic marker for IgAN and as a potential target for drug intervention. Nonetheless, further high-quality studies are needed to validate and elucidate the underlying mechanism.

Apart from the strong causality between Class Actinobacteria and IgAN, our study identified that seven additional microbiota have a nominal causal relationship with IgAN. Most of these microbiota have been reported in previous cross-sectional studies [30,31]. Although the Bonferroni-corrected test did not reveal a significant effect for these microbiota, this does not exclude the possibility of a connection between them and IgAN. The pathogenesis of IgAN is complex, and the interplay between the intestine and the kidney is diverse. Various bacteria participate in the crosstalk between the intestinal and renal axes, jointly regulating intestinal and renal homeostasis and playing a critical role in preventing the onset and progression of diseases [32]. These microorganisms, which have a nominal causality with multiple phenotypes, may also be involved in the key dialogue between the gut and kidney. Previous research has also indicated that a single bacterium may not be a key player in the pathogenesis of IgAN [30]. Comprehensive regulation of gut microbiota may represent a novel approach to intervene in IgAN in the future. Some comprehensive interventions, such as fecal microbiota transplantation, have demonstrated promising renal protective effects and reduced the accumulation of uremic toxins in animal models [33,34]. Clinical trials investigating the safety and efficacy of fecal microbiota transplantation in patients with IgAN are currently underway (NCT03633864). It is worth mentioning that with advancements in metagenomic sequencing, the complexity of the intestinal flora will be further explored, potentially enabling more accurate and comprehensive regulation of microbiota compared to fecal microbiota transplantation in the future. This development represents an important step forward in precision medicine targeting the gut–kidney axis. Understanding how these gut microbiota cooperate to induce IgAN may help us better comprehend the intricate dialogue and inspire future research on drugs targeting specific bacteria.

Regarding gut metabolites, our study only found that beta-hydroxybutyric acid is associated with a reduced risk of IgAN (OR: 0.97, 95% CI: 0.94–0.99, *p* = 0.037). It has been reported that beta-hydroxybutyric acid plays a role in the immune modulation of kidney diseases. Beta-hydroxybutyric acid can act as an inhibitor of oxidative stress and the NLRP3 inflammasome, reducing inflammatory responses and apoptosis [35]. Several studies have shown that beta-hydroxybutyric acid decreased the expression of caspase−1 and proinflammatory cytokines and improved renal injury in ischemia-reperfusion mice, indicating the role of beta-hydroxybutyric acid in blocking cell apoptosis and exerting a protective effect on the kidney [36]. Gong et al. found that beta-hydroxybutyric acid can regulate intestinal tight junction protein expression, promote IgA secretion, improve intestinal integrity, and prevent mucosal infection [37]. Although the Bonferroni-corrected test did not show a significant effect, beta-hydroxybutyric acid remains a potential therapeutic target for future IgAN intervention. Interestingly, some metabolites (e.g., trimethylamine N-oxide, *p*-cresyl sulfate) have been reported to be associated with the severity of immune kidney disease [14,38]; however, our study did not find such an association. This may be due to the following reasons: first, IgAN may exacerbate the accumulation of these metabolites, and these metabolites may also contribute to the worsening of kidney disease; however, it is unclear whether these metabolites are the root cause of IgAN, given that the majority of subject studies used cross-sectional analysis. Second, a variety of confounding factors could play a role in the pathogenesis of IgAN. Without the cooperation of these confounding factors, it is difficult to achieve statistical significance in gene prediction for a single metabolite.

It is worth noting that we did not find reverse causality between these specific microbiota and IgAN. This suggests that IgAN may not be the cause of these intestinal flora disorders, but rather the outcome of them. Our study established the direct and reverse causal links between specific microbiota and IgAN, something that prior studies had not done. This could help identify ways to use specific microbiota and metabolites as useful biomarkers for early disease detection for IgAN. This approach could potentially serve as an alternative to the prevalent use of kidney biopsies in diagnosing patients with IgAN, reducing the associated risks of bleeding, infection, and kidney injury. Furthermore, it may enable clinicians to more conveniently identify IgAN patients, allowing for earlier planning of diagnostic and treatment strategies, ultimately improving patient prognosis. Moreover, current treatment options for IgAN are quite limited. Understanding the underlying mechanisms between specific gut microbiota and IgAN could contribute to the development of targeted novel drug interventions for IgAN. This would not only help to circumvent the side effects associated with steroids and immunosuppressive agents but also significantly propel the advancement of precision medicine for IgAN.

It is equally important to acknowledge the limitations of our study. First, our clinical sample validation is still based on cross-sectional studies, and we have not been able to use cohort studies to verify the association between specific microbiota and IgAN. In addition, due to conditions and patient willingness, we failed to obtain more samples from patients with other glomerulonephritis (GN) conditions compared to those with IgAN. Some baseline characteristics of IgAN patients also varied from other GN patients, which may result in some bias. Although we found consistent results, the level of evidence is insufficient. Further cohort studies are warranted in the future. Secondly, although we identified the specific microbiota and metabolites that may be causally associated with IgAN, the interactive dialogue between the intestine and kidney is a complex and diverse process. It is necessary to understand how these specific microbiota and metabolites are synergistically involved in the pathogenesis of IgAN. Thirdly, at present, the metagenomic sequencing of intestinal flora is still improving. The complexity of the gut microbiota extends far beyond the 211 bacteria mentioned in this study. Further gene association studies and clinical research verification are necessary as gut microbiota sequencing technology advances.

## 5. Conclusions

In conclusion, our study provides valuable insights into the potential causal relationships between specific gut microbiota, metabolites, and IgAN. The identification of Actinobacteria as a strong causal factor, as well as the nominal causality of other microbiota, adds to our understanding of the intricate interplay between the gut and kidney in the development of IgAN. Moreover, the potential protective role of beta-hydroxybutyric acid offers an interesting avenue for future research and therapeutic intervention. While our findings contribute to the current knowledge base, further high-quality studies are necessary to validate these results and elucidate the underlying mechanisms. As precision medicine targeting the gut–kidney axis continues to evolve, a more comprehensive understanding of these complex relationships will pave the way for novel diagnostic and therapeutic strategies for IgAN and other immune kidney diseases.

## Figures and Tables

**Table 1:** Causal effects of gut microbiota and metabolites on IgAN.

Exposure	Odd Ratio	95% CI	*p*-Value
**Class.Actinobacteria**			
Weighted mode	1.27	1.05–1.53	0.036 *
Weighted median	1.2	1.03–1.41	0.024 *
IVW	1.2	1.07–1.36	0.0029 **
MR-Egger	1.15	0.83–1.61	0.42
**Family.Erysipelotrichaceae**			
Weighted mode	1.27	0.88–1.84	0.26
Weighted median	1.2	0.95–1.51	0.12
IVW	1.2	1.01–1.43	0.044 *
MR-Egger	1.34	0.61–2.98	0.5
**Order.Erysipelotrichales**			
Weighted mode	1.21	0.88–1.67	0.28
Weighted median	1.2	0.99–1.46	0.06
IVW	1.2	1.03–1.40	0.018 *
MR-Egger	1.32	0.68–2.58	0.43
**Genus.Lachnospira**			
Weighted mode	1.22	0.84–1.77	0.36
Weighted median	1.24	0.94–1.62	0.12
IVW	1.3	1.06–1.68	0.012 *
MR-Egger	1.18	0.34–4.09	0.81
**Genus.Parabacteroides**			
Weighted mode	0.81	0.61–1.08	0.21
Weighted median	0.81	0.64–1.02	0.07
IVW	0.79	0.67–0.94	0.0078 *
MR-Egger	0.93	0.55–1.55	0.78
**Genus.Butyrivibrio**			
Weighted mode	1.03	0.92–1.16	0.63
Weighted median	1.03	0.96–1.11	0.47
IVW	1.06	1.01–1.12	0.048 *
MR-Egger	1.11	0.86–1.42	0.44
**Genus.Phascolarctobacterium**			
Weighted mode	1.16	0.93–1.46	0.23
Weighted median	1.13	0.98–1.31	0.1
IVW	1.14	1.01–1.27	0.024 *
MR-Egger	1.1	0.67–1.82	0.71
**Genus.Ruminococcus**			
Weighted mode	0.83	0.63–1.05	0.21
Weighted median	0.84	0.71–1.01	0.055
IVW	0.86	0.76–0.98	0.02 *
MR-Egger	0.73	0.52–1.01	0.13
**Beta_hydroxybutyric acid**			
Weighted mode	0.98	0.93–1.02	0.3
Weighted median	0.97	0.93–1.01	0.17
IVW	0.97	0.94–0.99	0.037 *
MR-Egger	0.98	0.92–1.05	0.57

Bold: statistical significance; * nomial significant, ** significant. Abbreviation: IVE: Inverse variance weighted, CI: confidence interval, IgAN: immunoglobulin A nephropathy.

**Table 2:** Baseline characteristics of the included participants.

GROUP	HKC	IgAN	Others_GN	*p*-Value
N	10	10	5	
Age(years)	52.4 ± 21.2	52.8 ± 17.0	67.8 ± 16.5	0.205
SBP (mmHg)	129.2 ± 16.8	136.0 ± 15.9	132.1 ± 23.6	0.8
BMI (Kg/m2)	23.1 ± 3.9	25.0 ± 5.7	24.2 ± 3.5	0.715
HB (g/L)	14.2 ± 1.8	13.8 ± 1.8	11.0 ± 1.2	0.016 *
ALB (g/L)	4.3 ± 0.7	4.0 ± 0.5	3.9 ± 0.4	0.093
eGFR (ml/min/1.73 m2)	93.5 ± 1.9	55.5 ± 13.4	17.9 ± 10.3	<0.001 **
24 h proteinuria	0.1 ± 0.0	1.3 ± 1.6	3.1 ± 2.3	<0.001 **
Gender				0.867
Male	6 (60.0%)	7 (70.0%)	4 (80.0%)	
Female	4 (40.0%)	3 (30.0%)	1 (20.0%)	
Hypertension				0.025
No	6 (60.0%)	1 (10.0%)	0 (0.0%)	
Yes	4 (40.0%)	9 (90.0%)	5 (100.0%)	

Bold: statistical significance; *: *p* < 0.05; **: *p* < 0.01. Abbreviation: SBP: Systolic blood pressure; BMI: Body mass index; HB: Hemoglobin; ALB: Serum albumin; eGFR: Estimated glomerular filtration rate; HKC: Healthy kidney control; IgAN: Immunoglobulin A nephropathy; Others_GN: Non-IgAN glomerular disease.

## Data Availability

The datasets used and analyzed in this study are available from the first author and corresponding author on reasonable request.

## Supplementary Materials

The following supporting information can be downloaded at: https://www.mdpi.com/article/10.3390/nu15102407/s1, Figure S1: Scatter plots of causal estimates from genetically predicted metabolites on immunoglobulin A nephropathy; Figure S2: Funnel plots of causal estimates from genetically predicted metabolites on immunoglobulin A nephropathy. Figure S3: Leave-one-out plots of causal estimates from genetically predicted microbiota on immunoglobulin A nephropathy. Figure S4: Relationship between Actinobacteria and glomerular filtration rate and albuminuria in non-IgAN. Figure S5: The association of renal biopsy case score (MEST-C) with Acti abundance. Table S1: The instrumental variables of the gut microbiota. Table S2: The instrumental variables of the metabolites; Table S3: Mendelian randomized outliers and level pleiotropy test of exposure and outcome. Table S4: Inverse variance weighted analysis of the reverse causality between exposure and outcome. Table S5: Comparison of pathological scores (MEST-C scores) among groups with different relative abundance.

## References

[1] Schena F.P., Nistor I. (2018). Epidemiology of IgA Nephropathy: A Global Perspective. Semin. Nephrol..

[2] Canney M., Barbour S.J., Zheng Y., Coppo R., Zhang H., Liu Z.-H., Matsuzaki K., Suzuki Y., Katafuchi R., Reich H.N. (2021). Quantifying Duration of Proteinuria Remission and Association with Clinical Outcome in IgA Nephropathy. J. Am. Soc. Nephrol..

[3] Pattrapornpisut P., Avila-Casado C., Reich H.N. (2021). IgA Nephropathy: Core Curriculum 2021. Am. J. Kidney Dis..

[4] Coppo R. (2018). The Gut-Renal Connection in IgA Nephropathy. Semin. Nephrol..

[5] Cheung C.K., Rajasekaran A., Barratt J., Rizk D.V. (2021). An Update on the Current State of Management and Clinical Trials for IgA Nephropathy. J. Clin. Med..

[6] Barratt J., Rovin B.H., Cattran D., Floege J., Lafayette R., Tesar V., Trimarchi H., Zhang H. (2020). Why Target the Gut to Treat IgA Nephropathy?. Kidney Int. Rep..

[7] Rollino C., Vischini G., Coppo R. (2016). IgA nephropathy and infections. J. Nephrol..

[8] Seikrit C., Pabst O. (2021). The immune landscape of IgA induction in the gut. Semin. Immunopathol..

[9] Gesualdo L., Di Leo V., Coppo R. (2021). The mucosal immune system and IgA nephropathy. Semin. Immunopathol..

[10] Perše M., Večerić-Haler Ž. (2019). The Role of IgA in the Pathogenesis of IgA Nephropathy. Int. J. Mol. Sci..

[11] He J.W., Zhou X.J., Lv J.C., Zhang H. (2020). Perspectives on how mucosal immune responses, infections and gut microbiome shape IgA nephropathy and future therapies. Theranostics.

[12] Haniuda K., Gommerman J.L., Reich H.N. (2021). The microbiome and IgA nephropathy. Semin. Immunopathol..

[13] Han S., Shang L., Lu Y., Wang Y. (2022). Gut Microbiome Characteristics in IgA Nephropathy: Qualitative and Quantitative Analysis from Observational Studies. Front. Cell. Infect. Microbiol..

[14] Chen Y.-Y., Chen D.-Q., Chen L., Liu J.-R., Vaziri N.D., Guo Y., Zhao Y.-Y. (2019). Microbiome–metabolome reveals the contribution of gut–kidney axis on kidney disease. J. Transl. Med..

[15] Lauriero G., Abbad L., Vacca M., Celano G., Chemouny J.M., Calasso M., Berthelot L., Gesualdo L., De Angelis M., Monteiro R.C. (2021). Fecal Microbiota Transplantation Modulates Renal Phenotype in the Humanized Mouse Model of IgA Nephropathy. Front. Immunol..

[16] Aguilera M., Cerdà-Cuéllar M., Martínez V. (2015). Antibiotic-induced dysbiosis alters host-bacterial interactions and leads to colonic sensory and motor changes in mice. Gut Microbes.

[17] Davey S.G., Hemani G. (2014). Mendelian randomization: Genetic anchors for causal inference in epidemiological studies. Hum. Mol. Genet..

[18] Kurilshikov A., Medina-Gomez C., Bacigalupe R., Radjabzadeh D., Wang J., Demirkan A., Le Roy C.I., Garay J.A.R., Finnicum C.T., Liu X. (2021). Large-scale association analyses identify host factors influencing human gut microbiome composition. Nat. Genet..

[19] Rhee E.P., Ho J.E., Chen M.-H., Shen D., Cheng S., Larson M.G., Ghorbani A., Shi X., Helenius I.T., O’donnell C.J. (2013). A Genome-wide association study of the human metabolome in a community-based cohort. Cell Metab..

[20] Sakaue S., Kanai M., Tanigawa Y., Karjalainen J., Kurki M., Koshiba S., Narita A., Konuma T., Yamamoto K., Akiyama M. (2021). A cross-population atlas of genetic associations for 220 human phenotypes. Nat. Genet..

[21] Staley J.R., Blackshaw J., Kamat M.A., Ellis S., Surendran P., Sun B.B., Paul D.S., Freitag D., Burgess S., Danesh J. (2016). PhenoScanner: A database of human genotype-phenotype associations. Bioinformatics.

[22] Bowden J., Davey Smith G., Burgess S. (2015). Mendelian randomization with invalid instruments: Effect estimation and bias detection through Egger regression. Int. J. Epidemiol..

[23] Verbanck M., Chen C.-Y., Neale B., Do R. (2018). Detection of widespread horizontal pleiotropy in causal relationships inferred from Mendelian randomization between complex traits and diseases. Nat. Genet..

[24] Monteiro R.C., Berthelot L. (2021). Role of gut–kidney axis in renal diseases and IgA nephropathy. Curr. Opin. Gastroenterol..

[25] Sugurmar A.N.K., Mohd R., Shah S.A., Neoh H.-M., Cader R.A. (2021). Gut microbiota in Immunoglobulin A Nephropathy: A Malaysian Perspective. BMC Nephrol..

[26] Zhong Z., Tan J., Tan L., Tang Y., Qiu Z., Pei G., Qin W. (2020). Modifications of gut microbiota are associated with the severity of IgA nephropathy in the Chinese population. Int. Immunopharmacol..

[27] Li J., Cao Y., Lu R., Li H., Pang Y., Fu H., Fang G., Chen Q., Liu B., Wu J. (2021). Integrated Fecal Microbiome and Serum Metabolomics Analysis Reveals Abnormal Changes in Rats with Immunoglobulin A Nephropathy and the Intervention Effect of Zhen Wu Tang. Front. Pharmacol..

[28] Liu F., Xu X., Chao L., Chen K., Shao A., Sun D., Hong Y., Hu R., Jiang P., Zhang N. (2021). Alteration of the Gut Microbiome in Chronic Kidney Disease Patients and Its Association With Serum Free Immunoglobulin Light Chains. Front. Immunol..

[29] Wang Y., Zhao J., Qin Y., Yu Z., Zhang Y., Ning X., Sun S. (2022). The Specific Alteration of Gut Microbiota in Diabetic Kidney Diseases—A Systematic Review and Meta-Analysis. Front. Immunol..

[30] He J.-W., Zhou X.-J., Li Y.-F., Wang Y.-N., Liu L.-J., Shi S.-F., Xin X.-H., Li R.-S., Falchi M., Lv J.-C. (2021). Associations of Genetic Variants Contributing to Gut Microbiota Composition in Immunoglobin A Nephropathy. Msystems.

[31] Shah N.B., Nigwekar S.U., Kalim S., Lelouvier B., Servant F., Dalal M., Krinsky S., Fasano A., Tolkoff-Rubin N., Allegretti A.S. (2021). The Gut and Blood Microbiome in IgA Nephropathy and Healthy Controls. Kidney360.

[32] Yang T., Richards E.M., Pepine C.J., Raizada M.K. (2018). The gut microbiota and the brain–gut–kidney axis in hypertension and chronic kidney disease. Nat. Rev. Nephrol..

[33] Barba C., Soulage C.O., Caggiano G., Glorieux G., Fouque D., Koppe L. (2020). Effects of Fecal Microbiota Transplantation on Composition in Mice with CKD. Toxins.

[34] Bian J., Liebert A., Bicknell B., Chen X.-M., Huang C., Pollock C.A. (2022). Faecal Microbiota Transplantation and Chronic Kidney Disease. Nutrients.

[35] Luo S., Yang M., Han Y., Zhao H., Jiang N., Li L., Chen W., Li C., Yang J., Liu Y. (2022). β-Hydroxybutyrate against Cisplatin-Induced acute kidney injury via inhibiting NLRP3 inflammasome and oxidative stress. Int. Immunopharmacol..

[36] Tajima T., Yoshifuji A., Matsui A., Itoh T., Uchiyama K., Kanda T., Tokuyama H., Wakino S., Itoh H. (2019). β-hydroxybutyrate attenuates renal ischemia-reperfusion injury through its anti-pyroptotic effects. Kidney Int..

[37] Gong Y., Jin X., Yuan B., Lv Y., Yan G., Liu M., Xie C., Liu J., Tang Y., Gao H. (2021). G Protein-Coupled Receptor 109A Maintains the Intestinal Integrity and Protects Against ETEC Mucosal Infection by Promoting IgA Secretion. Front. Immunol..

[38] Lau W.L., Savoj J., Nakata M.B., Vaziri N.D. (2018). Altered microbiome in chronic kidney disease: Systemic effects of gut-derived uremic toxins. Clin. Sci..

