# Supplementary material for: The Effects of Specific Gut Microbiota and Metabolites on IgA Nephropathy—Based on Mendelian Randomization and Clinical Validation
Source: Nutrients. 2023 May 22;15(10):2407. doi: 10.3390/nu15102407 (PMC10221929; doi:10.3390/nu15102407)
Supplement: Supplementary file 1 [file nutrients-15-02407-s001.zip › Supple Table S4.pdf]

Supplement Table S4. Inverse variance weighted analysis of the reverse causality between exposure and outcome.

Exposure	Outcome	β (95%CI)	p value
	**Class**		
IgAN	Actinobacteria	0.0002(-0.0008, 0.0012)	0.69
	**Family**		
IgAN	Erysipelotrichaceae	0.0002(-0.00075, 0.00118)	0.7
	**Genus**		
IgAN	Butyrivibrio	-0.0007(-0.0024, 0.0023)	0.96
IgAN	Parabacteroides	-0.0005(-0.00015, 0.00048)	0.93
IgAN	Ruminococcus	0.0003(-0.0007, 0.0012)	0.499
IgAN	Phascolarctobacterium	-0.0005(-0.002, 0.0009)	0.42
IgAN	Lachnospira	0.0004(-0.0006, 0.0014)	0.44
	**Order**		
IgAN	Erysipelotrichales	0.0002(-0.00085, 0.0012)	0.7
	**Metabolic**		
IgAN	Beta_hydroxybutyric acid	-0.003(-0.006, 0.002)	0.43

Abbreviations: IgAN: immunoglobulin A nephropathy
