# Supplementary material for: The Effects of Specific Gut Microbiota and Metabolites on IgA Nephropathy—Based on Mendelian Randomization and Clinical Validation
Source: Nutrients. 2023 May 22;15(10):2407. doi: 10.3390/nu15102407 (PMC10221929; doi:10.3390/nu15102407)
Supplement: Supplementary file 1 [file nutrients-15-02407-s001.zip › Supple Table S5.pdf]

Supplement Table S5. Comparison of pathological scores (MEST-C scores) among groups with different relative abundance.

Group	Low abundance group	High abundance group	p-value
**Mesangial.hypercellularity**			0.524
M0	3 (75.00%)	2 (33.33%)	
M1	1 (25.00%)	4 (66.67%)	
**Endocapillary_hypercellularity**			0.524
EO	3 (75.00%)	2 (33.33%)	
E1	1 (25.00%)	4 (66.67%)	
**Segmental glomerulosclerosis/adhesion**			0.2
S0	4 (100.00%)	3 (50.00%)	
S1	0 (0.00%)	3 (50.00%)	
**Tubular atrophy/interstitial fibrosis**			0.467
T0	4 (100.00%)	3 (50.00%)	
T1	0 (0.00%)	1 (16.67%)	
T2	0 (0.00%)	2 (33.33%)	
**Crescents**			1
C0	4 (100.00%)	5 (83.33%)	
C1	0 (0.00%)	1 (16.67%)	
