# Supplementary material for: The Effects of Specific Gut Microbiota and Metabolites on IgA Nephropathy—Based on Mendelian Randomization and Clinical Validation
Source: Nutrients. 2023 May 22;15(10):2407. doi: 10.3390/nu15102407 (PMC10221929; doi:10.3390/nu15102407)
Supplement: Supplementary file 1 [file nutrients-15-02407-s001.zip › Supplement Table S3.pdf]

Supplement Table S3. Mendelian randomized outliers and level pleiotropy test of exposure and outcome

Group	Exposure	Outcome	Cochran's Q-derived P value	MR-PRESSO-Global-test-derived P value	MR-Egger intercept-derived P value
Class	*Actinobacteria*	IgAN	0.96	0.95	0.79
Family	*Erysipelotrichaceae*	IgAN	0.62	0.63	0.79
Genus	*Lachnospira*	IgAN	0.70	0.74	0.89
	Parabacteroides	IgAN	0.74	0.78	0.58
	Butyrivibrio	IgAN	0.55	0.55	0.71
	Phascolarctobacterium	IgAN	0.85	0.86	0.91
	Ruminococcus	IgAN	0.69	0.70	0.37
Order	*Erysipelotrichales*	IgAN	0.87	0.83	0.42
Metabolite	*Beta_hydroxybutyric acid*	IgAN	0.27	0.32	0.73
