# Supplementary figures and images for: The Effects of Specific Gut Microbiota and Metabolites on IgA Nephropathy—Based on Mendelian Randomization and Clinical Validation
Source: Nutrients. 2023 May 22;15(10):2407. doi: 10.3390/nu15102407 (PMC10221929; doi:10.3390/nu15102407)

# MR Test

- Inverse variance weighted
- MR Egger
- Weighted median

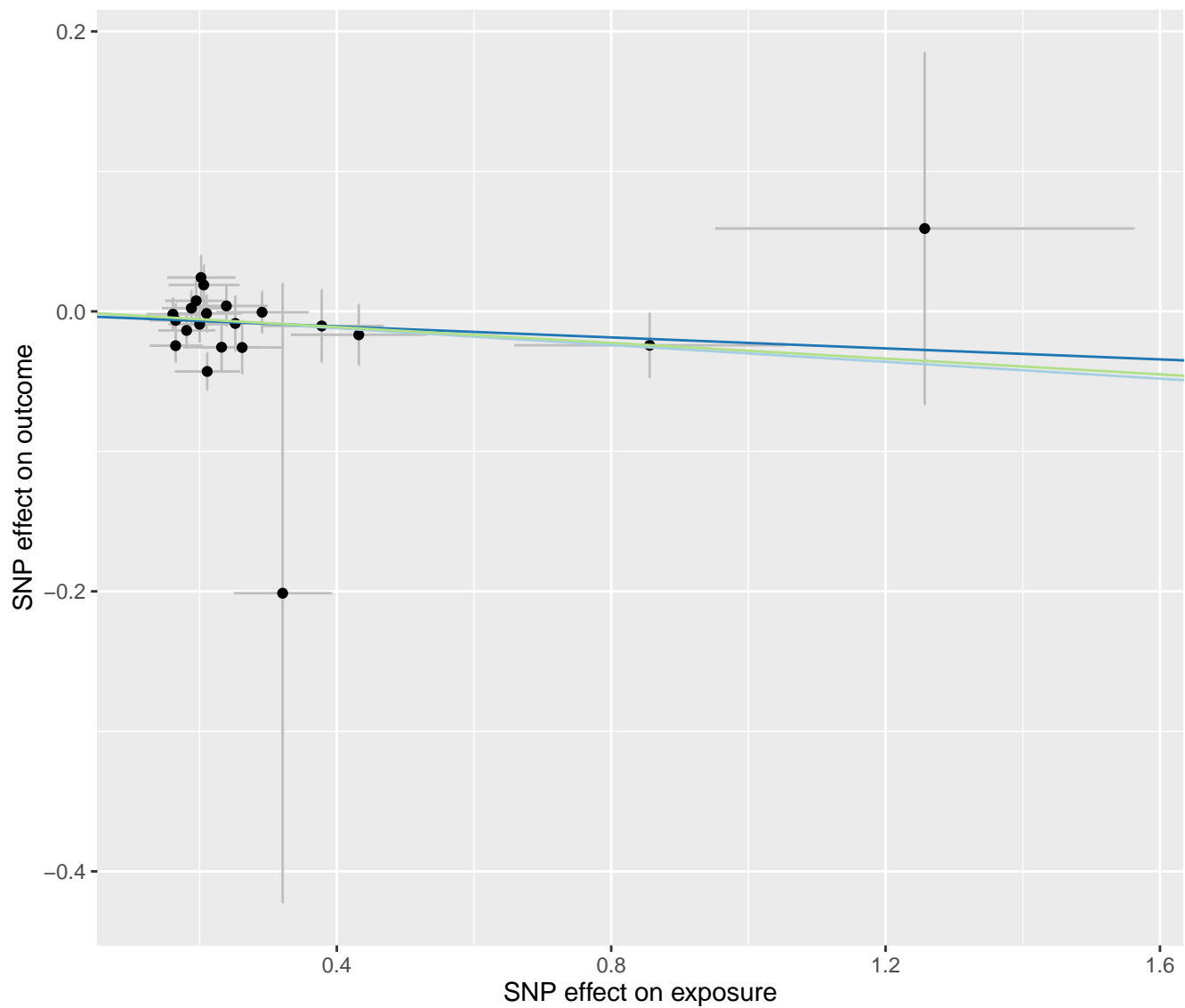

Supplement: Supplementary file 1 [file nutrients-15-02407-s001.zip › Sup Figure S1.pdf]

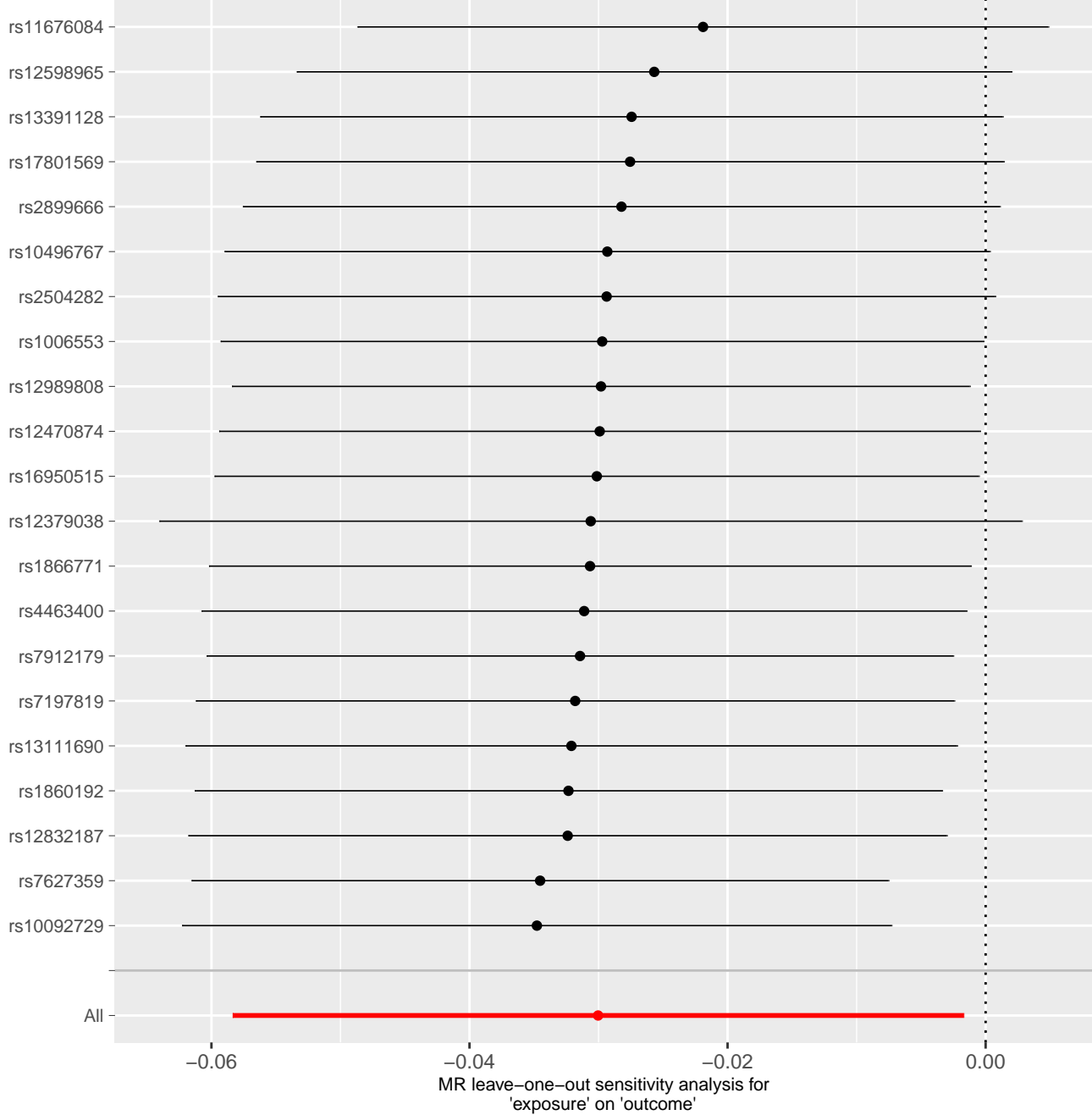

Supplement: Supplementary file 1 [file nutrients-15-02407-s001.zip › Sup Figure S3.pdf]

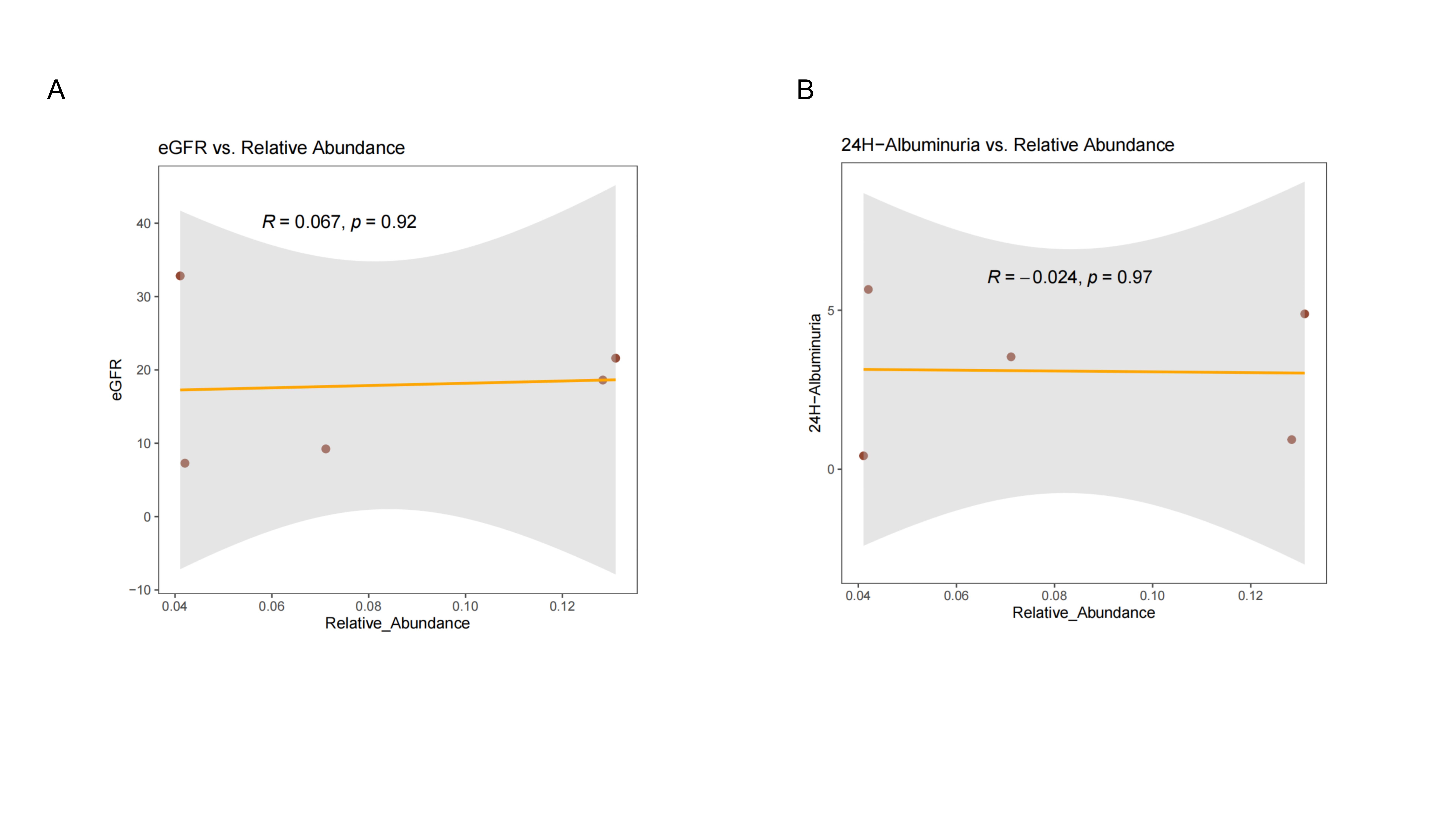

Supplement: Supplementary file 1 [file nutrients-15-02407-s001.zip › Sup Figure S4.jpg]

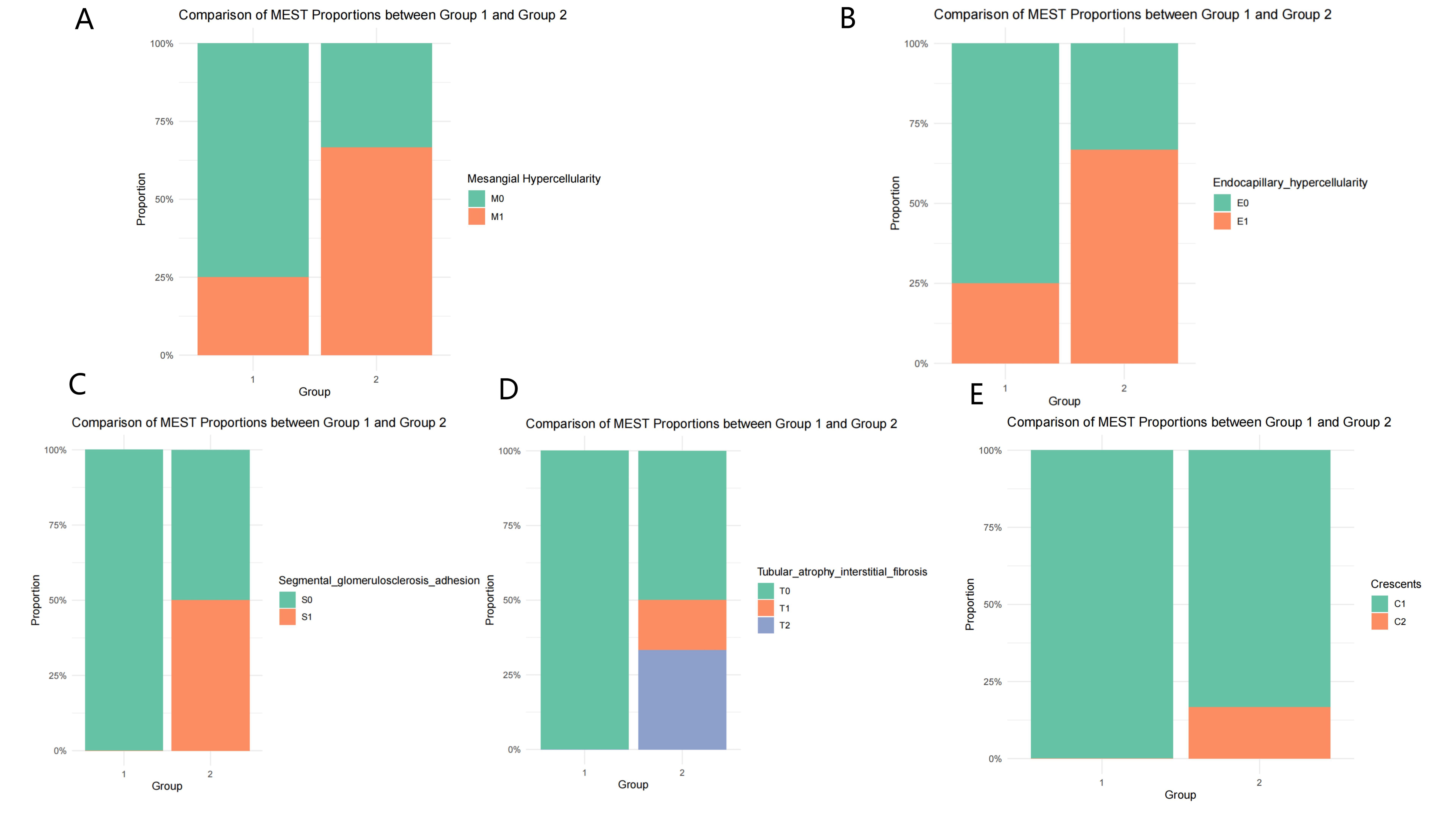

Supplement: Supplementary file 1 [file nutrients-15-02407-s001.zip › Sup Figure S5.jpg]
