# Supplementary material for: The Effects of Specific Gut Microbiota and Metabolites on IgA Nephropathy—Based on Mendelian Randomization and Clinical Validation
Source: Nutrients. 2023 May 22;15(10):2407. doi: 10.3390/nu15102407 (PMC10221929; doi:10.3390/nu15102407)
Supplement: Supplementary file 1 [file Sup Figure S2.pdf › Sup Figure S2.pdf]

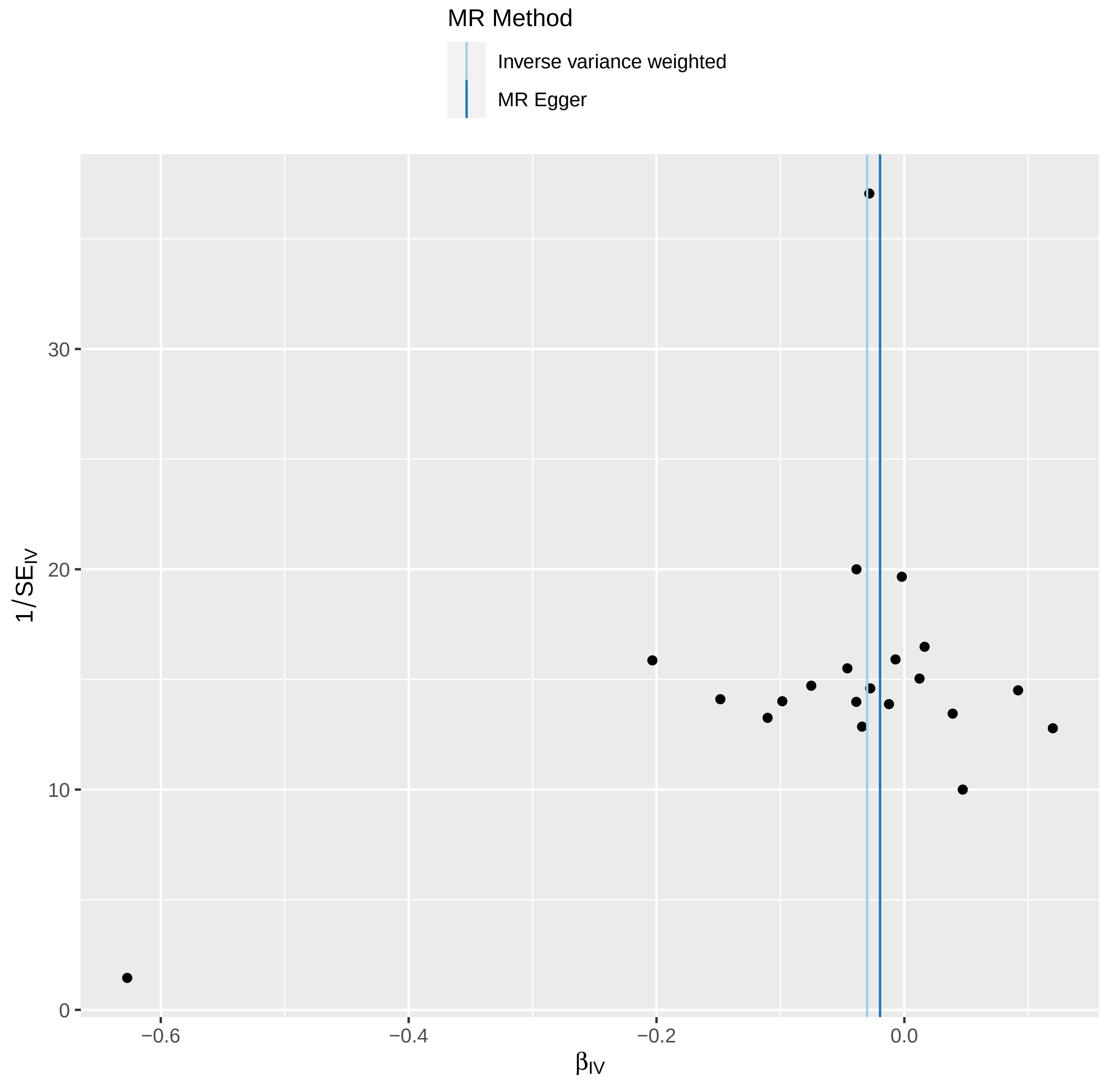
MR Method
Inverse variance weighted
MR Egger
30
20
10
0
1/SE_IV
−0.6
−0.4
−0.2
0.0
β_IV
